# Neural progenitor cells isolated from the subventricular zone present hemichannel activity and form functional gap junctions with glial cells

**DOI:** 10.3389/fncel.2015.00411

**Published:** 2015-10-13

**Authors:** Rocío Talaverón, Paola Fernández, Rosalba Escamilla, Angel M. Pastor, Esperanza R. Matarredona, Juan C. Sáez

**Affiliations:** ^1^Departamento de Fisiología, Facultad de Biología, Universidad de SevillaSevilla, Spain; ^2^Departamento de Fisiología, Pontificia Universidad Católica de Chile, Santiago de Chile, Chile and Instituto Milenio, Centro Interdisciplinario de Neurociencias de ValparaísoChile

**Keywords:** subventricular zone, microglia, astrocytes, gap junctions, hemichannels, dye coupling, dye uptake

## Abstract

The postnatal subventricular zone (SVZ) lining the walls of the lateral ventricles contains neural progenitor cells (NPCs) that generate new olfactory bulb interneurons. Communication via gap junctions between cells in the SVZ is involved in NPC proliferation and in neuroblast migration towards the olfactory bulb. SVZ NPCs can be expanded *in vitro* in the form of neurospheres that can be used for transplantation purposes after brain injury. We have previously reported that neurosphere-derived NPCs form heterocellular gap junctions with host glial cells when they are implanted after mechanical injury. To analyze functionality of NPC-glial cell gap junctions we performed dye coupling experiments in co-cultures of SVZ NPCs with astrocytes or microglia. Neurosphere-derived cells expressed mRNA for at least the hemichannel/gap junction channel proteins connexin 26 (Cx26), Cx43, Cx45 and pannexin 1 (Panx1). Dye coupling experiments revealed that gap junctional communication occurred among neurosphere cells (incidence of coupling: 100%). Moreover, hemichannel activity was also detected in neurosphere cells as evaluated in time-lapse measurements of ethidium bromide uptake. Heterocellular coupling between NPCs and glial cells was evidenced in co-cultures of neurospheres with astrocytes (incidence of coupling: 91.0 ± 4.7%) or with microglia (incidence of coupling: 71.9 ± 6.7%). Dye coupling in neurospheres and in co-cultures was inhibited by octanol, a gap junction blocker. Altogether, these results suggest the existence of functional hemichannels and gap junction channels in postnatal SVZ neurospheres. In addition, they demonstrate that SVZ-derived NPCs can establish functional gap junctions with astrocytes or microglia. Therefore, cell-cell communication via gap junctions and hemichannels with host glial cells might subserve a role in the functional integration of NPCs after implantation in the damaged brain.

## Introduction

Neurogenesis in the adult rodent brain persists during adulthood in two main neurogenic zones, the subgranular zone of the dentate gyrus in the hippocampus, and the subventricular zone (SVZ) lining the walls of the lateral ventricles. The postnatal and adult SVZ contains neural progenitor cells (NPCs) that give rise to transit-amplifying intermediate progenitors, which after several divisions differentiate into neuroblasts. SVZ neuroblasts migrate along the rostral migratory stream towards the olfactory bulb where they generate new interneurons (Gage, 2000; Anderson, 2001; Alvarez-Buylla et al., 2002). Gap junction intercellular communication has been described to occur in the SVZ at homocellular and heterocellular contacts (Menezes et al., 2000; Lacar et al., 2011). This type of cellular communication provides a mechanism for the coordination of metabolic and electrical activities (Bruzzone et al., 1996; Kumar and Gilula, 1996) that might play an active role in shaping the specific behavior of the SVZ cell population.

Gap junction channels are composed of two hemichannels, one on each of the communicating cells. Hemichannels in vertebrates can be constituted by two types of proteins: connexins or pannexins. There are 21 different connexins known to date, which differ primarily in their C-terminal domain, molecular weight and tissue specificity (Willecke et al., 2002). Among all connexins, Cx26 (molecular weight of about 26 kDa), Cx43 and Cx45 are expressed by NPCs of different sources (Nadarajah et al., 1997; Duval et al., 2002; Cina et al., 2007; Freitas et al., 2012; Khodosevich et al., 2012). Specifically, Cx45 is involved in the modulation of proliferation and differentiation of NPCs from the postnatal SVZ (Khodosevich et al., 2012). Although the majority of connexin hemichannels are docked to function as gap junction channels, unapposed hemichannels have also been documented on the surface of different cell types (Chen et al., 2005; Retamal et al., 2006). These channels, when open, serve as a conduit between the intracellular space and the external environment allowing uptake of metabolic substrates and release of autocrine and paracrine signals. The presence and possible functional role of open connexin hemichannels in postnatal SVZ NPCs remains to be elucidated. Hemichannels formed by pannexins, in contrast to those formed by connexins, might not form gap junction channels (Dahl and Locovei, 2006; D’hondt et al., 2009; MacVicar and Thompson, 2010). It has been recently reported that postnatal SVZ NPCs and their immature neuronal progeny express pannexin 1 (Panx1) as large pore channels that mediate the release of ATP, which has been proposed to regulate NPC proliferation by interaction with purinergic P2 receptors (Wicki-Stordeur et al., 2012).

NPCs of the SVZ can be isolated and amplified *in vitro* in the form of floating aggregates termed neurospheres (Carpenter et al., 1999; Vescovi et al., 1999; Gage, 2000). NPCs derived from SVZ neurospheres provide an interesting cell population to be used for transplantation purposes in different types of brain lesions, not only because of their capacity to integrate into the host tissue, contributing to the possible replacement of damaged cells, but also because of several bystander capacities such as tissue trophic support and immune regulation (Ben-Hur, 2008). The ability of implanted NPCs to integrate and exert benefitial effects to the lesioned host tissue depends on local interactions created in the microenvironment of the site of grafting in which communication between implanted and host cells are crucial (Martino and Pluchino, 2006; Martino et al., 2011). For instance, Jäderstad et al. (2010) reported that an early and essential step in the functional integration of grafted NPCs is cell-cell coupling via gap junctions with host neurons that permits exogenous NPCs to influence directly host network activity. In line with this, we have recently reported that implanted NPCs after mechanical brain injury establish gap junctional communication with host astrocytes and microglia (Talaverón et al., 2014).

As a further step, we have aimed to analyze whether gap junctions formed between NPCs and astrocytes or microglial cells are functional. For that purpose, we have performed dye coupling experiments in neurospheres and in co-cultures of SVZ-derived NPCs and primary astrocytes or microglia. Our results show that cells from SVZ neurospheres present hemichannel activity and are coupled via gap junctions. In addition, we describe that gap junctional communication is established between neurosphere-derived cells and astrocytes or microglia *in vitro*.

## Materials and Methods

### Animals

Experiments were conducted on 1-day and 7-day postnatal Sprague-Dawley rats obtained from the animal facilities of the Faculty of Biological Sciences of the Pontificia Universidad Católica de Chile. All procedures were in accordance with the institutional guidelines and were approved by the Bioethic and Biosecurity Committee of the Pontificia Universidad Católica de Chile.

### Cell Culture Reagents

Dulbecco’s modified Eagle’s medium (DMEM), minimum essential medium (MEM), DMEM-F-12 1:1 medium (DF-12), ${\text{HCO}}_{3}^{-}$ free F-12 medium, B-27 supplement, HEPES, GlutaMAX^™^, phosphate buffered saline (PBS), PBS without Ca^2+^ and Mg^2+^, trypsin (0.5%-EDTA 5 mM), trypsin (2.5%), penicillin/streptomycin 100X, horse serum and fetal bovine serum were purchased from GIBCO (Life Technologies, Carlsbad, CA, USA). Bovine pancreas DNaseI, poly-D-lysine, DiI, La^3+^, Lucifer yellow (LY), ethidium bromide and octanol were purchased from Sigma-Aldrich (St. Louis, MO, USA). Epidermal growth factor (EGF) was purchased from Peprotech (London, UK) and basic fibroblast growth factor (FGF-2) from Millipore (Darmstadt, Germany). Coverslips were purchased from Marienfeld Laboratory Glassware (Lauda-Könighshofen, Germany). All plastic culture flasks and dishes were from Sarstedt Inc. (Nümbrecht, Germany).

### Neural Progenitor Cell Culture

NPCs were isolated from the SVZ of 7-day postnatal rats and were expanded in the form of neurospheres essentially as described before (Talaverón et al., 2013). Briefly, the lateral walls of the lateral ventricles were removed and enzymatically dissociated with 1 mg/ml trypsin at 37°C for 15 min. The tissue was then centrifuged at 150×g for 5 min, rinsed in DF-12 and centrifuged again in the same conditions. Then, cells were resuspended in DF-12 and mechanically disaggregated with a fire-polished Pasteur pipette. The dissociated cells were centrifuged, resuspended in neurosphere growth medium consisting in DF-12 added with B-27 supplement, GlutaMAX^™^, 100 units/ml penicillin and 100 μg/ml streptomycin, 20 ng/ml EGF and 10 ng/ml FGF-2, and maintained in an atmosphere of 5% CO_2_, at 37°C. After 1–2 days, cell aggregates known as neurospheres were formed. Cells were subcultured 48 h after isolation and then every 3–4 days. Cells used in the experiments were obtained from neurospheres after a minimum of two and a maximum of six subcultures. For dye coupling and dye uptake experiments, neurospheres were slightly disaggregated and plated on poly-D-lysine-treated coverslips.

### Detection of Cx26, Cx43, Cx45, and Panx1 mRNAs

Relative levels of Cx26, Cx43, Cx45, and Panx1 mRNA were determined by RT-PCR. Total RNA was isolated from 700,000 neurosphere-derived cells using TRIzol reagent (Ambion) according to the manufacturer’s instructions. A RNA (10 μg) aliquot was treated with RQ1 RNase free-DNase (Promega) for 30 min at 37°C and purified with a second round of TRIzol. cDNA was synthesized from 2 μg total RNA using SuperScript^™^ First-Strand Synthesis System for RT-PCR (Invitrogen) according to the manufacturer instructions using random hexamers and the oligo (dT) primer provided with the kit. For detection, PCR reactions for Cx26, Cx43, Cx45, and Panx1 were performed with 1 μL of cDNA in 25 μl of reaction and contained 1.5 mM MgCl_2_, 160 μM dNTPs, 240 nM of each forward and reverse primers, 1X Green GoTaq reaction buffer (Promega) and 1 unit of GoTaq DNA polymerase (Promega). For the control gene GAPDH, PCR reaction was similar as for target genes with the exception of the primer concentration, which was 160 nM for each one. Sequences of PCR primers were as follows, Cx26 (forward, GGAGATGAGCAAGCCGATTT; reverse, GAAGAAGATGCTGGTGGTGTAG), Cx43 (forward, ATCCTTACCACGCCACCA; reverse, GCTAATGGCTGGAGTTCATGTC), Cx45 (forward AAAGAGCAGAGCCAACCA; reverse, GAATGGTCCCAAACCCTAGAT), Panx1 (forward, GAGATATCCGAAAGCCACTTCA; reverse, GGCGTACACTAGGAGGTTAATG) and GAPDH (forward, ACCACAGTCCATGCCATCAC; reverse, TCCACCACCCTGTTGCTGTA). PCR products were resolved in 1.5% agarose/1× TAE buffer electrophoresis (Invitrogen) and stained with ethidium bromide (0.5 μg/ml) for visualization.

### Primary Culture of Astrocytes and Microglia

Primary cultures of astrocytes and microglia were prepared from 1-day postnatal rat neocortex. After removal of meninges, cortices were dissected, minced in small pieces and incubated at 37°C for 30 min in trypsin (0.5%) and EDTA (5 mM) prepared in MEM supplemented with antibiotics (100 units/ml penicillin and 100 μg/ml streptomycin). After removal of the enzyme solution, tissue was triturated in dissociation medium (MEM, 10% horse serum and antibiotics) added with 0.01 mg/ml DNase I from bovine pancreas with the use of a Pasteur pipette. Dissociated cells were pelleted, resuspended in dissociation medium and plated on plastic culture flasks at 37°C in a 5% CO_2_/95% air atmosphere. After 24 h, the medium was removed and replaced by growth medium (DMEM supplemented with 10% fetal bovine serum and antibiotics).

For astrocyte culture, the medium was replaced every other day. Five days after plating, cells were detached by treatment with trypsin (0.5%) and EDTA (5 mM) in PBS without Ca^2+^ and Mg^2+^. After detachment, cells were collected, centrifuged and plated on 15 mm diameter coverslips with growth medium at a density of 20,000 cells/coverslip. More than 95% of cells showed immunoreactivity for glial fibrillary acidic protein.

Flasks reserved for microglia culture were kept for 10–15 days without medium renewal to allow microglia proliferation in astrocyte-conditioned medium. Cultures were then rigorously agitated for 30 min in an orbital shaker (Lab-Line Instruments) at 70 rpm and 37°C to detach cells adhering to the astrocyte monolayer. Thereafter, detached cells were collected, plated on 15 mm glass coverlips (20,000 cells/coverslip) and maintained in growth medium. Close to 99% of the cells obtained after this procedure were immunopositive for the ED-1 antigen but were negative for glial fibrillary acidic protein, indicating a very high enrichment in microglia (Eugenín et al., 2001).

### Co-culture of Neurosphere-Derived Cells and Glial Cells

Coverslips having confluent primary cultures of astrocytes or microglia were labeled with a lipophilic dye before co-culturing them with neurosphere-derived cells. For that purpose, cells were incubated with DiI (5 μM) for 15 min at 37°C and washed three times with PBS. Thirty minutes later, neurospheres were subjected to a brief mechanical disaggregation and plated on coverslips containing the DiI-labeled astrocytes or microglial cells (20,000 neurosphere-derived cells/coverslip). Co-cultures were maintained at 37°C in a 5% CO_2_/95% air atmosphere with neurosphere growth medium for a minimum of 1 h before experiments.

### Dye Coupling

The functional state of gap junctions was evaluated as previously described (Martínez and Sáez, 1999) in neurosphere-derived cells and in co-cultures of neurosphere-derived cells with astrocytes or microglia. Single NPCs were iontophoretically microinjected with a glass micropipette filled with 75 mM LY (5% w/v in 150 mM LiCl). Dye coupling index was calculated as the mean number of cells to which the dye spread occurred in 3 min. All microinjections were performed in ${\text{HCO}}_{3}^{-}$ free F-12 medium buffered with 10 mM HEPES (pH 7.4) containing 200 μM La^3+^ to avoid cell leakage of the microinjected dye via hemichannels. Dye coupling was assessed in the absence and in the presence of 750 μM octanol. Fluorescent cells were observed using a Nikon inverted microscope equipped with epifluorescence illumination (Xenon arc lamp) and Nikon B filter to LY (excitation wavelength 450–490 nm; emission wavelength above 520 nm) and XF34 filter to DiI fluorescence (Omega Optical, Inc., Brattleboro, VT, USA). Photomicrographs were obtained using a CCD monochrome camera (CFW-1310M; Scion; Frederick, MD, USA). Five to ten experiments were performed for every type of culture and dye coupling was tested by microinjecting a minimum of 10 cells per experiment.

### Dye Uptake

Hemichannel activity in neurosphere-derived cells was evaluated by using the ethidium (Etd) bromide uptake method (Schalper et al., 2008). Neurospheres plated on 25 mm poly-D-lysine-treated coverslips were transferred to a 30 mm dish, coated with a thin layer of vaseline to immobilize the coverslips. Then, cells were washed twice with a recording solution (in mM: NaCl (148); KCl (5); CaCl_2_ (1.8); MgCl_2_ (1); glucose (5); HEPES (5), *pH* = 7.4) containing 5 μM Etd. Basal fluorescence intensity from selected regions of the cells was recorded for 5 min, and cells were subsequently exposed for 5 min to a recording solution without divalent cations (divalent cation-free solution, DCFS). To confirm that Etd uptake was mediated by hemichannels, the blocker La^3+^ (200 μM) was added at the end of each recording (Schalper et al., 2008) and fluorescence was recorded for another 5 min. Dye uptake was recorded in an Olympus BX51WI upright microscope using a 40× water immersion objective (Melville), and equipped with the image acquisition system Q Imaging, model Retiga 13001, fast-cooled monochromatic digital camera (12 bit) (Qimaging, Burnaby, BC, Canada). Images were captured every 30 s (*exposure time* = 30 ms, *gain* = 0.5). Metafluor software (version 6.2R5, Universal Imaging Co., Downingtown, PA, USA) was used for off-line image analysis and fluorescence quantification. For data representation and calculation of uptake rates, the average of three independent background fluorescence intensity measurements (FB, expressed as arbitrary units, AU) was subtracted from the fluorescence intensity in each cell (F1). Results of this calculation (F1-FB), including at least 10 cells per experiment, were averaged and plotted against time (expressed in minutes). Dye uptake rates were calculated with Microsoft Excel software and expressed as AU/min. Microscope and camera settings remained constant in all experiments.

### Statistics

Data are presented as mean ± standard error of the mean (SEM) and were analyzed in Sigma Plot 11 (Systat Software) by using the one-way analysis of variance (ANOVA) followed by a Tukey’s *post hoc* test or Student’s *t*-test, as appropriate. Differences were considered significant at a level of *p* < 0.05.

## Results

### Cells Obtained from Neurospheres Express mRNAs for Hemichannel and Gap Junction Proteins

**Figure 1:**
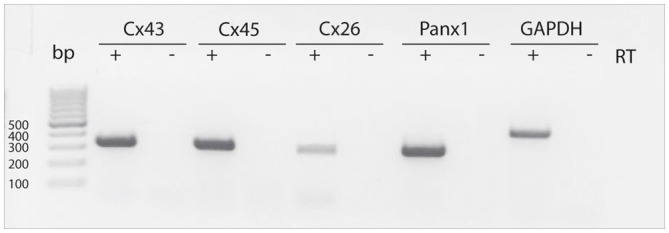
**Detection of Cx43, Cx45, Cx26 and Panx1 mRNAs in SVZ neurosphere-derived cells.** Two μg of total RNA from neurosphere-derived cells was analyzed by RT-PCR with specific primers designed to detect the presence of Cx43, Cx45, Cx26 and Panx1 mRNAs. Detection of the housekeeping GAPDH mRNA was included as a loading control. Lane 1, 100-bp DNA ladder. Lanes labeled—RT are negative controls without reverse transcriptase enzyme. A representative agarose gel from 4 independent experiments is shown.

RT-PCR measurements were performed to detect mRNAs for different connexins (Cx43, Cx45, Cx26) as wells as for Panx1 in neurospheres obtained from the postnatal rat SVZ. We sought to detect the mRNAs of these proteins based on previous reports showing evidence for a role of these connexins on NPC proliferation (Cheng et al., 2004; Kunze et al., 2009; Khodosevich et al., 2012). RT-PCR revealed the presence of Cx43, Cx45, Cx26 and Panx1 mRNA in neurosphere-derived cells (Figure 1).

### Cell Coupling in Neurosphere-Derived Cells

Neurospheres were slightly disaggregated and plated on 15 mm poly-D-lysine-treated coverslips. Both non-disaggregated neurospheres and small clusters of about 5–25 cells were observed after plating which will be generally referred to as neurosphere-derived cells. LY was microinjected in a single cell and the dye transfer to adjacent cells was evaluated three minutes later. All microinjections were performed short after plating (maximum 1 h), when most neurosphere-derived cells remained undifferentiated.

**Figure 2:**
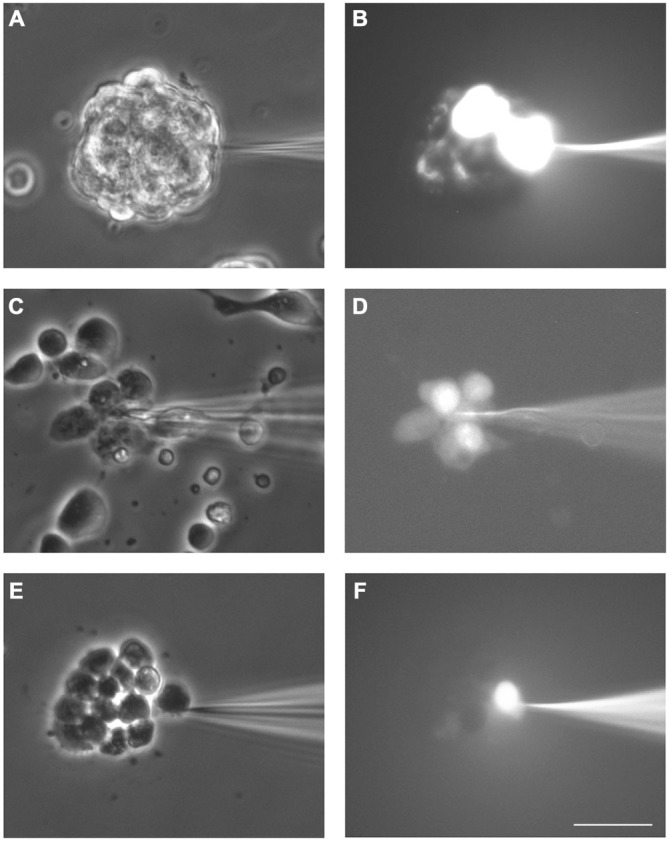
**Dye coupling between cells in SVZ neurospheres.** Examples of dye coupling 3 min after Lucifer yellow (LY) microinjection into a cell of a non-disaggregated neurosphere **(A, B)** or into a neurosphere-derived cell **(C, D)**. After addition of the gap junction blocker octanol (750 μM), microinjected LY did not spread to neighboring neurosphere-derived cells **(E, F)**. **(A,C)** and **(E)** show the corresponding phase-contrast views of the fields of the representative pictures of LY microinjections shown in **(B,D)** and **(F)**, respectively. Bar: 50 μm.

All the LY microinjections performed in neurosphere-derived cells led to dye transfer to adjacent cells, therefore, the incidence of coupling was 100% (data from 52 cells from five different experiments). Figures 2A,B show an example of LY transfer to neighboring cells in a non-disaggregated neurosphere. Dye coupling also occurred in the small groups of neurosphere-derived cells (see example in Figures 2C,D; in Figure 2D the cell microinjected with LY shows the highest intensity of fluorescence). In the presence of the gap junction opening inhibitor octanol (750 μM), dye coupling was completely abolished (an example is shown in Figures 2E,F).

The index of coupling in neurosphere-derived cells, calculated as the mean number of cells to which the dye was transferred in positive cases, was 3.0 ± 0.3 cells. This index was significantly higher than that obtained in neurosphere cultures after addition of octanol (0 ± 0 cells; Student’s *t*-test) so we can conclude that intercellular dye transfer in neurosphere-derived cells occurred indeed through gap junctional communication.

### Connexin-Mediated Hemichannel Activity in Neurosphere-Derived Cells

Hemichannel activity was tested in neurosphere-derived cells by analyzing time-lapse measurements of Etd uptake in different experimental conditions. Both non-disaggregated neurospheres and small clusters of 5–25 cells from slightly disaggregated neurospheres were analyzed.

**Figure 3:**
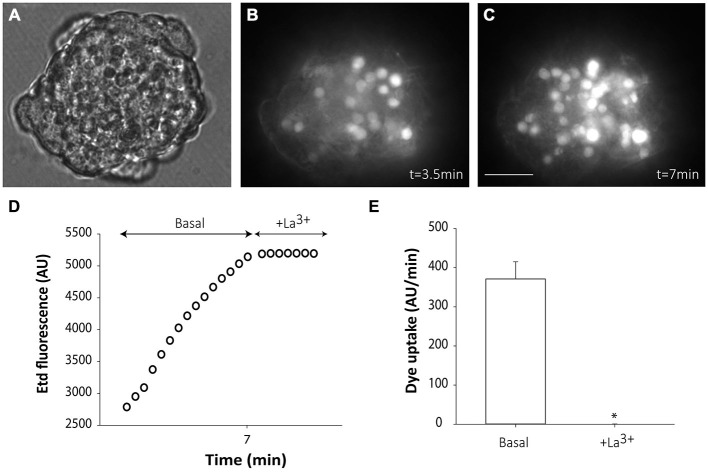
**Floating SVZ neurospheres present high connexin hemichannel activity.** Representative fluorescence images of a non-disaggregated floating neurosphere incubated in a solution containing 5 μM ethidium (Etd) for 3.5 min **(B)** and 3.5 min later **(C)**. **(A)** shows the corresponding phase-contrast image. Bar: 25 μm. **(D)**: Time-lapse Etd uptake (in arbitrary units, AU) under basal conditions (Basal; first 7 min) and after addition of the connexin hemichannel blocker 200 μM La^3+^ (+La^3+^, following 3.5 min). Each point corresponds to the mean of 20 cells from one experiment. **(E)**: Etd uptake rates (in AU/min) in cells of neurospheres under basal conditions and after La^3+^ addition (+La^3+^). Data are presented as mean ± SEM of three independent experiments. *p < 0.05 compared to basal conditions (Student’s *t* test).

Cells from non-disaggregated neurospheres showed evident Etd uptake under basal conditions (Figures 3A–E). Etd uptake occurred through connexin hemichannels since addition of the connexin hemichannel blocker La^3+^ to the bath solution completely abolished it (Figures 3D,E). Etd uptake in basal conditions was only evident short after neurosphere plating (up to ~1 h) and disappeared as cells adhered to the substrate (data not shown). This suggests that, in floating neurospheres, hemichannels are open in resting conditions and are closed or removed from the cell surface once cells begin to adhere to a substrate, a condition that initiates cell differentiation towards neural phenotypes.

**Figure 4:**
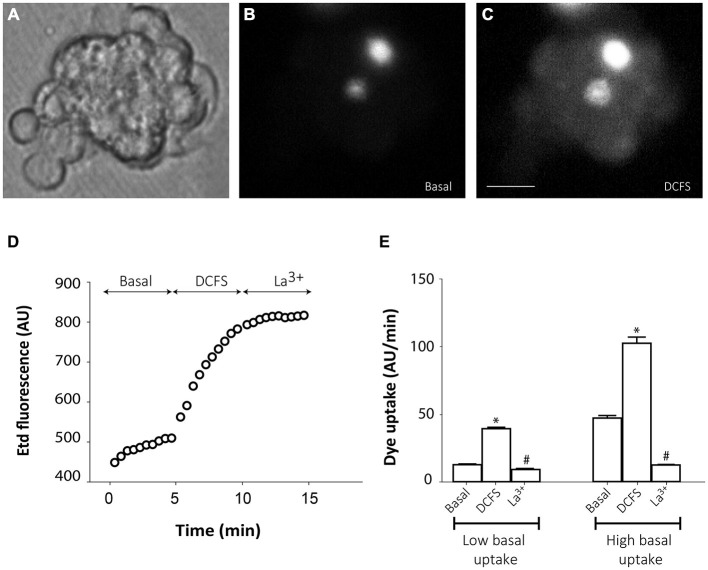
**Connexin hemichannel activity in SVZ neurosphere-derived cells.** Representative fluorescence images of a small cluster of neurosphere-derived cells incubated in a solution containing 5 μM ethidium (Etd) under basal conditions **(B)** and after bathing with divalent cation-free solution (DCFS) to increase the open probability of connexin hemichannels **(C)**. **(A)** shows the corresponding phase-contrast image. Brightest cells in B are cells with high basal Etd uptake in contrast to the majority of neurosphere-derived cells that presented very low basal Etd uptake. Bar: 25 μm. **(D)**: Time-lapse Etd uptake (in arbitrary units, AU) under basal conditions (Basal; first 5 min), after incubation in DCFS (DCFS, following 5 min) and after addition of the connexin hemichannel blocker 200 μM La^3+^ (La^3+^, following 5 min). Each point corresponds to the mean of 10 cells with low basal Etd uptake from one experiment. **(E)**: Etd uptake rates (in AU/min) in neurosphere-derived cells measured under basal conditions (Basal), after incubation in DCFS (DCFS) and after La^3+^ addition (La^3+^) in low basal Etd uptake cells and in high basal Etd uptake cells. Data are presented as mean ± SEM from three independent experiments. *p < 0.05 compared to basal conditions. ^#^*p* < 0.05 compared to DCFS condition (one-way ANOVA followed by Tukey’s *post hoc* test).

However, in the majority of cells from small groups of slightly disaggregated neurospheres, Etd uptake was very low under basal conditions (Figures 4A,B,D), which probably indicates that mechanical disaggregation induces hemichannel closing or hemichannel removal from the cell surface. Only few cells in every analyzed cell cluster showed high basal Etd uptake (brightest cells in Figure 4B). Replacement of the extracellular saline solution by DCFS to increase the open probability of connexin hemichannels (Schalper et al., 2008), induced a progressive increase in Etd uptake in all cells (Figures 4C,D,E), indicating that hemichannels were indeed located at the cells surface but presented very low open probability. Addition of the connexin hemichannel blocker La^3+^ (200 μM) in this condition significantly inhibited Etd uptake in all neurosphere-derived cells (Figures 4D,E). Inducible opening of hemichannels in neurosphere-derived cells was only achieved shortly after plating. After 2–3 h of plating, change to DCFS did not induce Etd uptake, indicating a loss of activatable hemichannel probably due to removal from the cell membrane as a result of cell adhesion (data not shown).

### Cell Coupling between Neurosphere-Derived Cells and Astrocytes

**Figure 5:**
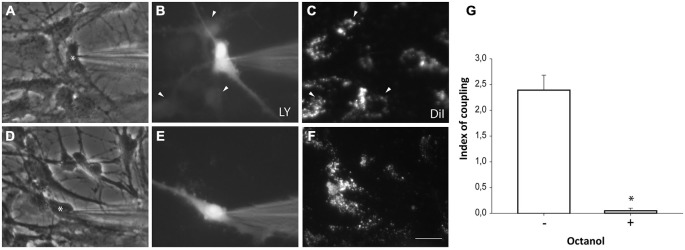
**Neurosphere-derived cells and astrocytes establish gap junctional communication.** Photomicrographs showing examples of LY microinjections in co-cultures of neurosphere-derived cells and primary astrocytes. Astrocytes were labeled with the lipophilic dye DiI before the co-culture to allow their identification. LY was microinjected in neurosphere-derived cells and the transfer to adjacent DiI-labeled cells was evaluated three minutes later. **(A)**: Phase-contrast view of the fields shown in **(B,C)**. The asterisk denotes the cell microinjected with LY. **(B)**: LY microinjection in a neurosphere-derived cell and dye transfer to adjacent cells (arrowheads). **(C)**: DiI-labeled cells of the same field shown in **(B)**. Cells that received the dye are indicated with arrowheads. **(D)**: Phase-contrast view of the fields shown in **(E,F)**. The asterisk denotes the cell microinjected with LY. **(E)**: LY microinjection in a neurosphere-derived cell after addition of the gap junction blocker octanol (750 μM). A lack of dye transfer to adjacent cells is observed. **(F)**: DiI-labeled cells of the same field shown in **(E)**. Bar: 50 μm. **(G)**. Index of coupling, calculated as the mean number of cells that received LY in every injection, in co-cultures of neurosphere-derived cells and astrocytes in control conditions (−) and after addition of octanol to the extracellular saline solution (+). Results are the mean ± SEM of 96 cells from nine independent experiments). *p < 0.05 compared to control conditions (Student’s *t* test).

Neurosphere-derived NPCs were plated on astrocyte monolayers to analyze gap junctional communication between both cell populations. Astrocytes were pre-labeled with the lipophilic dye DiI to distinguish them from neurosphere-derived NPCs. One hour after setting the co-culture, dye coupling was evaluated by microinjecting LY in non-labeled NPCs and analyzing the dye transfer to adjacent DiI-labeled astrocytes. Most injected NPCs were coupled to astrocytes (incidence of coupling of 91.0 ± 4.7%; 96 cells from nine independent experiments; Figures 5A–C) and LY injected in one cell diffused to a mean of 2.4 ± 0.3 adjacent astrocytes (index of coupling) (Figure 5G). Blockade of gap junctions with octanol (750 μM) prevented diffusion of LY from injected NPCs to adjacent astrocytes (Figures 5D–F,G).

### Cell Coupling between Neurosphere-Derived Cells and Microglia

**Figure 6:**
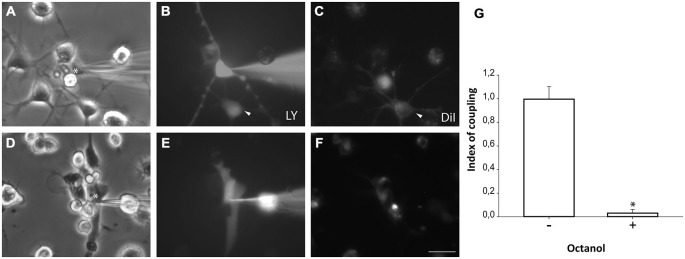
**Neurosphere-derived cells and microglia establish gap junctional communication.** Photomicrographs showing examples of LY microinjections in co-cultures of neurosphere-derived cells and primary microglia. Microglial cells were labeled with the lipophilic dye DiI before the co-culture to allow their identification. LY was microinjected in neurosphere-derived cells and the transfer to adjacent DiI-labeled cells was evaluated. **(A)**: Phase-contrast view of the fields shown in **(B,C)**. The asterisk denotes the cell microinjected with LY. **(B)**: LY microinjection in a neurosphere-derived cell and dye transfer to an adjacent cell (arrowhead). **(C)**: DiI-labeled cells of the same field shown in **(B)**. An example of a DiI-labeled cell that received LY is indicated with an arrowhead. **(D)**: Phase-contrast view of the fields shown in **(E,F)**. The asterisk denotes the cell microinjected with LY. **(E)**: LY microinjection in a neurosphere-derived cell after addition of the gap junction blocker octanol (750 μM). A lack of dye transfer to adjacent cells is observed. **(F)**: DiI-labeled cells of the same field shown in **(E)**. Bar: 50 μm. **(G)**. Index of coupling, calculated as the mean number of cells that received LY in every injection, in co-cultures of neurosphere-derived cells and microglia in control conditions (−) and after addition of octanol to the medium (+). Results are the mean ± SEM of 99 cells from 10 independent experiments). *p < 0.05 compared to control conditions (Student’s *t* test).

Microglial cells from primary cultures were also pre-labeled with DiI before NPCs were plated on them. One hour after co-culture, LY microinjections were performed in non-labeled NPCs and diffusion to DiI-labeled microglial cells was analyzed. Experiments revealed that NPCs also coupled to microglial cells (Figures 6A–C,G). However, a lower incidence of coupling with respect to astrocyte co-cultures was observed (71.9 ± 6.7%; 99 cells from 10 independent experiments). The index of dye coupling was 1.0 ± 0.1, also significantly lower than the obtained in NPCs-astrocyte co-cultures (*p* < 0.05; Student’s *t* test). Again, octanol prevented LY transfer from injected cells to neighbouring cells indicating that blocking gap junctions eliminated dye coupling between NPCs and microglia (Figures 6D–F,G).

## Discussion

In this study, we show evidence of hemichannel activity in NPCs from postnatal rat SVZ neurospheres. In addition, we demonstrate the existence of functional heterocellular gap junctions between neurosphere-derived cells and astrocytes or microglia. As NPCs are widely used for transplantation purposes in brain lesion models, we propose that this type of direct NPC-glial cell communication might take place in the host tissue with relevant roles for survival and integration of NPCs after implantation.

We have previously described that NPCs from postnatal rat SVZ express Cx43 *in vitro* and after implantation in the lesioned brain (Talaverón et al., 2014). In the current study, we have demonstrated that SVZ neurosphere-derived NPCs also express the mRNA for Cx26, Cx43, Cx45 and Panx1. Indeed, different roles for these hemichannel/gap junction proteins have been described in the SVZ neurogenic niche. For instance, Cx45 modulates the proliferation of transit-amplifying cells and neuroblasts in the postnatal SVZ by inducing cell cycle reentry via ATP signaling (Khodosevich et al., 2012). Also Panx1, expressed by NPCs in the SVZ, regulates NPC proliferation through the release of ATP (Wicki-Stordeur et al., 2012). Cx43 expression by NPCs from embryonic tissue is involved in their maintenance in a proliferative state (Cheng et al., 2004; Lemcke and Kuznetsov, 2013). Therefore, all results published to date point to a determinant role of the communication mediated by these hemichannel/gap junction proteins in the proliferation rate of this population of progenitor cells.

In the neurogenic postnatal SVZ, NPCs and niche astrocytes form microdomains of gap junctional communication networks (Lacar et al., 2011). The specific role of this functional connectivity has not yet been elucidated although it has been proposed that astrocytes could shape the behavior of NPCs through functional coupling and calcium waves. Direct homocellular communication via gap junctions among NPCs also takes place in the postnatal SVZ that is involved in cell migration throughout the rostral migratory stream (Menezes et al., 2000; Marins et al., 2009). We show, in this study, that the ability of postnatal SVZ NPCs to form functional gap junctions remains when they are isolated and are grown as neurospheres. Dye coupling in neurospheres occurred in the presence of extracellular La^3+^, that blocks connexin hemichannels (D’hondt et al., 2009), and it was abolished by octanol, a gap junction blocker that does not significantly affect pannexin hemichannels (D’hondt et al., 2009). This indicates that the coupling took place through gap junctions and was not due to cell leakage and reuptake via pannexin or connexin hemichannels.

Autocrine/paracrine communication via open hemichannels might also occur in SVZ neurospheres since, as described before, they express different connexins as well as Panx1. We have demonstrated that cells from floating non-disaggregated neurospheres present evident hemichannel activity mediated by connexin hemichannels since it was abolished by La^3+^, which specifically blocks connexin and not pannexin hemichannels (Pelegrin and Surprenant, 2006). Interestingly, hemichannel activity ceases when neurospheres adhere to a substrate, a condition that initiates cell differentiation towards neural phenotypes (Talaverón et al., 2013). This suggests that signaling through hemichannels may intervene on the maintainance of SVZ NPCs in an undifferentiated and proliferative state within the neurosphere. To our knowledge, this is the first study describing connexin hemichannel activity in postnatal SVZ neurospheres although its precise physiological role will have to be solved in future experiments.

NPCs isolated from the postnatal SVZ can form gap junctions after implantation in the mechanically-lesioned brain (Talaverón et al., 2014). Remarkably, gap junctions were identified not only among implanted NPCs, but also between NPCs and host astrocytes and less frequently with host microglia at the lesion site (Talaverón et al., 2014). In co-culture experiments of SVZ-derived NPCs with astrocytes, we have demonstrated that NPCs form functional gap junctions with astrocytes with more than a 90% incidence of coupling. As it was mentioned before, NPCs and astrocytes establish cellular networks of gap junctional communication in the SVZ neurogenic niche (Lacar et al., 2011) so, it is not surprising that the ability to form gap junctions between these two cell types remains *in vitro*. Therefore, it is quite feasible that gap junctions formed between NPCs implanted in the damaged brain and host astrocytes could be functional and have specific roles in NPC integration and in NPC-induced beneficial effects.

The demonstration of gap junctional communication within microglial cells and between microglia and neurons has been documented in *in vitro* experiments. Thereby, two independent groups have demonstrated that microglial cells can form functional gap junctions among them after activation with pro-inflammatory conditions (Eugenín et al., 2001; Garg et al., 2005). Also (Dobrenis et al., 2005) showed that in co-cultures, microglia can communicate with neurons via gap junctions. In contrast, in a recent study performed *in vivo*, both in normal and in diseased animals, the authors failed to identify any evidence of dye transfer between microglial cells or between microglia and neurons (Wasseff and Scherer, 2014). It is important to point out that we have previously identified at ultrastructural level the existence of gap junctions between implanted NPCs and host microglial cells in animals lesioned by axotomy (Talaverón et al., 2014). Now, we report here for the first time evidence of dye coupling between NPCs and microglia *in vitro*. The ability of NPCs to form functional gap junctions with microglia can be both physiologically and pathologically relevant. In healthy conditions, close proximity between microglial cells and NPCs has been documented in the SVZ neurogenic niche (Goings et al., 2006; González-Pérez et al., 2012; Mosher et al., 2012). Indeed, microglia and SVZ NPCs establish a bilateral cross-talk that can affect both microglial activation state and also NPC proliferation and differentiation (Mathieu et al., 2010; González-Pérez et al., 2012; Mosher et al., 2012; Shigemoto-Mogami et al., 2014). We propose here that direct communication via gap junctions between microglia and NPCs might also occur physiologically in the SVZ neurogenic niche with roles in the physiology of both cell types. In pathological conditions, possible scenarios for NPCs-microglia interactions include the use of NPC implants in lesioned brain with microglia activation. The inflammatory response triggered by host microglial cells is known to preserve implanted NPCs in an undifferentiated state in which they can promote CNS tissue healing by the secretion of immunomodulatory and neuroprotective molecules (Martino and Pluchino, 2006). As we have demonstrated *in vitro* direct NPC-microglia coupling, we raise the possibility that functional gap junctions between implanted NPCs and host microglia in the lesioned tissue might also intervene on restorative mechanisms induced by the implants.

Altogether our results demonstrate that postnatal SVZ NPCs cultured as neurospheres present functional hemichannels and gap junctions. In addition, neurosphere-derived cells can establish gap junctional communication with astrocytes and with microglia *in vitro*. Gap junctional communication between NPCs and glial cells might be involved in the behavior of NPCs both in their natural niche and also after implantation in the injured brain.

## Author Contributions

AMP, ERM and JCS designed research. RT, PF, RE and JCS performed research. RT, PF, RE, AMP, ERM and JCS analyzed data. ERM and JCS wrote the paper.

## Conflict of Interest Statement

The authors declare that the research was conducted in the absence of any commercial or financial relationships that could be construed as a potential conflict of interest.
